# Influence of frame and probe paths on the frame effect

**DOI:** 10.1167/jov.24.7.11

**Published:** 2024-07-16

**Authors:** Stuart Anstis, Patrick Cavanagh

**Affiliations:** 1 Department of Psychology, University of California San Diego, La Jolla, CA, USA; 2 Department of Psychology, Glendon College, Toronto, Ontario, Canada; 3 CVR, York University, North York, Ontario, Canada

**Keywords:** motion, motion-induced position shift

## Abstract

Moving frames produce large displacements in the perceived location of flashed and continuously moving probes. In a series of experiments, we test the contributions of the probe's displacement and the frame's displacement on the strength of the frame's effect. In the first experiment, we find a dramatic position shift of flashed probes whereas the effect on a continuously moving probe is only one-third as strong. In Experiment 2, we show that the absence of an effect for the static probe is a consequence of its perceptual grouping with the static background. As long as the continuously present probe has some motion, it appears to group to some extent with the frame and show an illusory shift of intermediate magnitude. Finally, we informally explored the illusory shifts seen for a continuously moving probe when the frame itself has a more complex path. In this case, the probe appears to group more strongly with the frame. Overall, the effects of the frame on the probe demonstrate the outcome of a competition between the frame and the static background in determining the frame of reference for the probe's perceived position.

## Introduction

**Figure 1.**
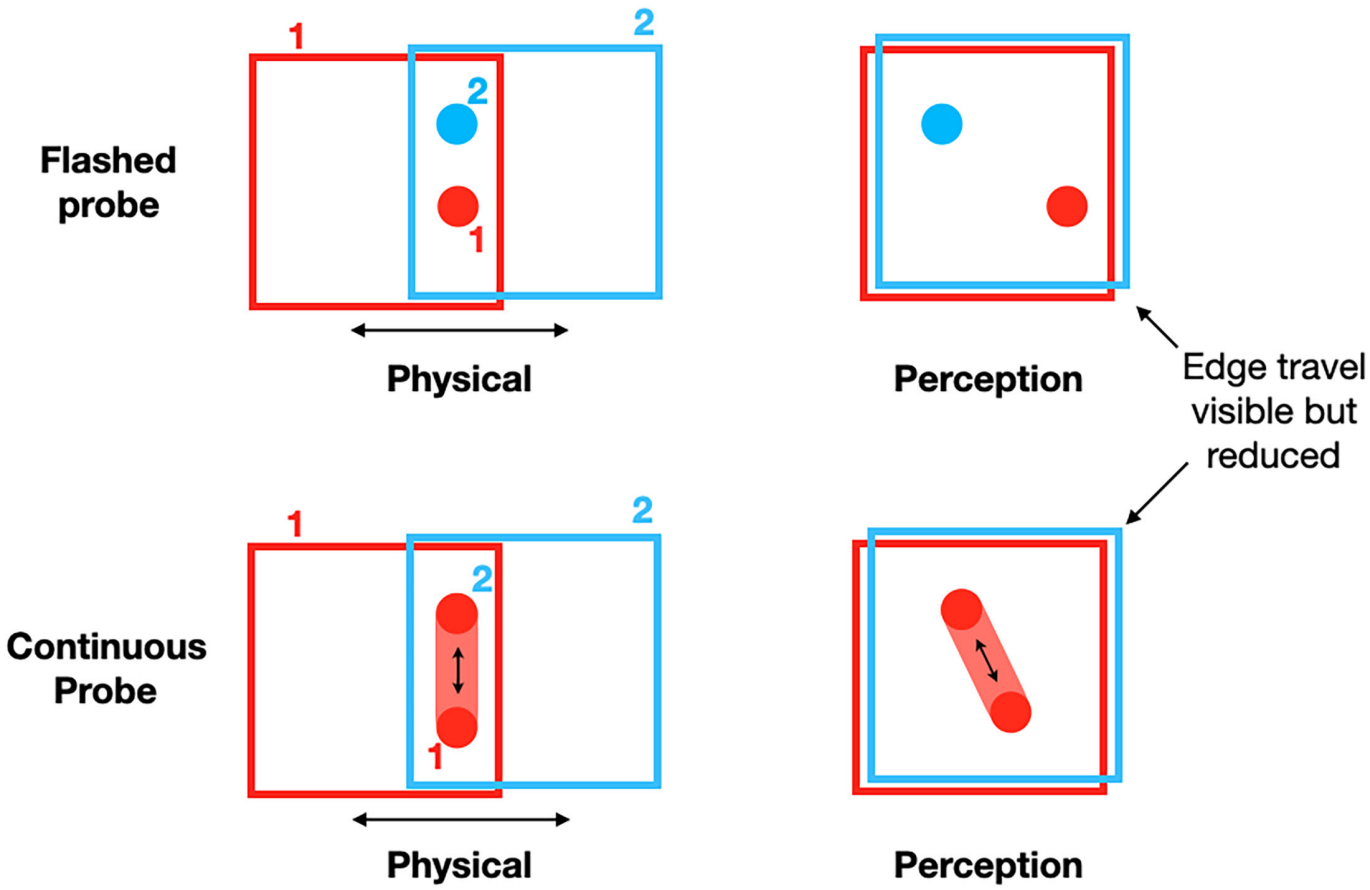
(Top left) Flashed condition: the two probes are vertically aligned on the screen (Movie 1). (Top right) The two probes appear strongly offset despite their physical alignment. (Bottom left) Continuous condition. The single probe moves up and down vertically (Movie 2). (Bottom right) This is the classic induced-motion stimulus (Wallach et al. 1978), and the perceived direction is a combination of the frame and probe directions.

Frames and backgrounds have very powerful influences on vision, changing our judgment of coordinates in the world such as “up” (Asch & Witkin, 1948; Morgan, Grant, Melmoth & Solomon, 2015) or “straight ahead” (Roelofs, 1935; Matin & Fox, 1989). When the frame is in motion, it changes the apparent direction of an object within the frame (Duncker, 1929; Johansson, 1950; Wallach, 1959). This earlier literature on frame effects typically examined static (Duncker, 1929) or continuously moving probes (Wallach, Bacon, & Schulman, 1978). Recently, we have shown that moving frames give far larger effects for flashed than for continuous probes (Özkan, Anstis,’t Hart, Wexler, & Cavanagh, 2021; Cavanagh et al., 2022). These large effects for flashed tests had previously been reported by Duncker (1929) and Wong and Mack (1981). When the probes are flashed, the illusory offsets can be as large as the frame's displacement, as if the flashes were seen in the frame's coordinates and the frame were not moving. For example, in Movie 1, when the blue disc flashes, it is near the left edge of the frame and when the red disk flashes it is near the right edge of the frame. Both flashes are actually vertically aligned on the display, but they are often seen with blue on the left and red on the right, their respective positions relative to the frame (Figure 1).

**Movie 1.** The frame effect with flashed probes. Click on the movie to open it in another window. An outline square moves left and right and red and blue disks are flashed alternately at each motion reversal. Although the two disks are always vertically aligned on the display, the blue disk may appear to have shifted to the left of the red disk. These are the positions the two disks have relative to the frame when they flash: the blue near the left edge and the red near the right edge. But it is the frame that has moved, not the disks. Movie is available on the journal website.

**Movie 2.** The frame effect with a continuous moving probe. Click on the movie to open it in a new window. The outline square moves left and right while a single red disk moves continuously. At the top on the right are two markers that are adjusted to match the perceived angle of the probe's motion. (1) In the first segment of the video, the probe moves up and down, initially alone on the screen. Once the frame is present, the probe's path may appear tilted to the left. This is the classic induced motion described by Wallach et al. (1978). (2) In the second segment, the probe moves with a large physical tilt to the right that makes the motion strictly up and down relative to the frame. However, rather than appearing to move vertically, its path remains tilted to the right although less so than its physical path. Movie is available on the journal website.

**Figure 2.**
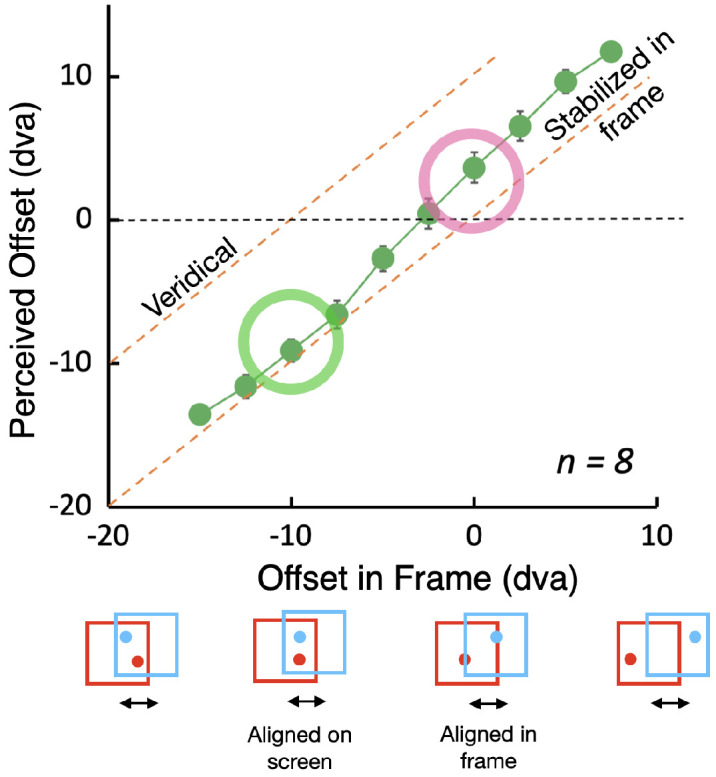
Flashed probes. Perceived offset as a function of physical offset in the frame. Path length was 10 degrees of visual angle (dva), frame size 20 dva. Data that fall along the top, oblique dashed line would be seen veridically with no effect of the frame. Data that fall along the bottom oblique line are seen in frame coordinates. When flashes were vertically aligned on the screen (green circle), they were seen to be separated by about 10 dva, the distance the frame traveled. When they were vertically aligned relative to the frame but separated horizontally by 10 dva on the screen, they appeared to be midway between veridical and stabilized in the frame (magenta circle). Error bars show ±1 standard error when larger than the data symbols.

**Movie 3.** The frame effect with flashed probes aligned in frame coordinates. Double click on the movie to open it in a new window. The markers to the top right are adjusted to match the perceived angle between the two probes. Both probes are vertically centered in the frame when they flash, but because the frame moves, they are widely spaced on the display, as can be seen when the frame momentarily fades out. When the frame is present, the two probes appear closer to vertical alignment than to their physical orientation (Figure 2, magenta circle). Movie is available on the journal website.

**Figure 3.**
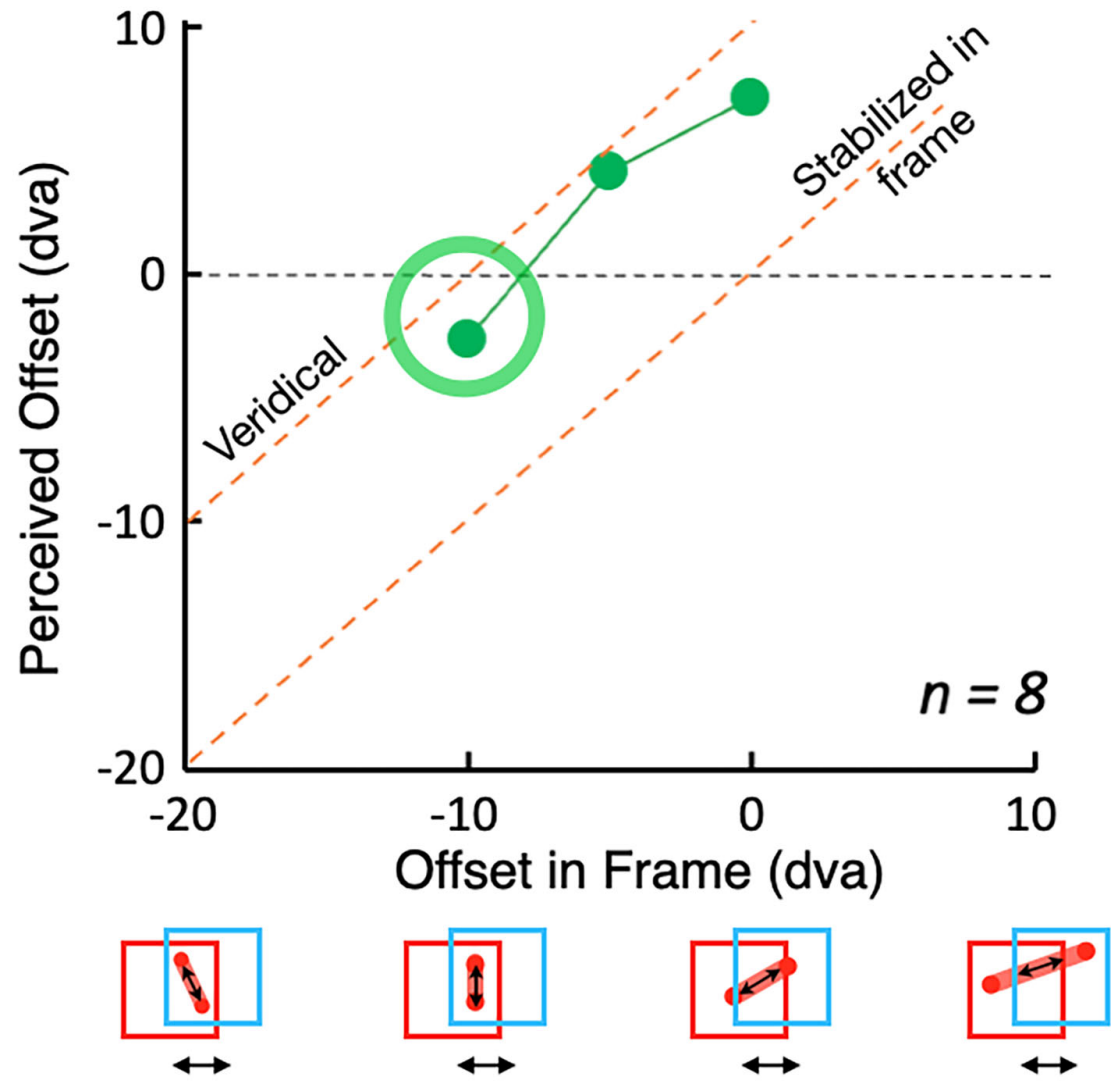
Continuous probe motion. Perceived offset as a function of physical offset of the probe in the frame. The perceived orientation of the motion path was tilted counterclockwise relative to its true orientation. When the motion path was vertical on the screen, its path appeared rotated away from vertical, with the top end shifted 2.6 degrees of visual angle (dva) horizontally from the bottom end (Movie 2). This was less than was seen for the flashed probes in Figure 2 where the top flash was seen shifted on average 9.1 dva away from the bottom flash. Error bars would show ±1 standard error, but they are all smaller than the data symbols.

**Movie 4.** All 10 stimulus conditions. Movie is available on the journal website.

**Figure 4.**
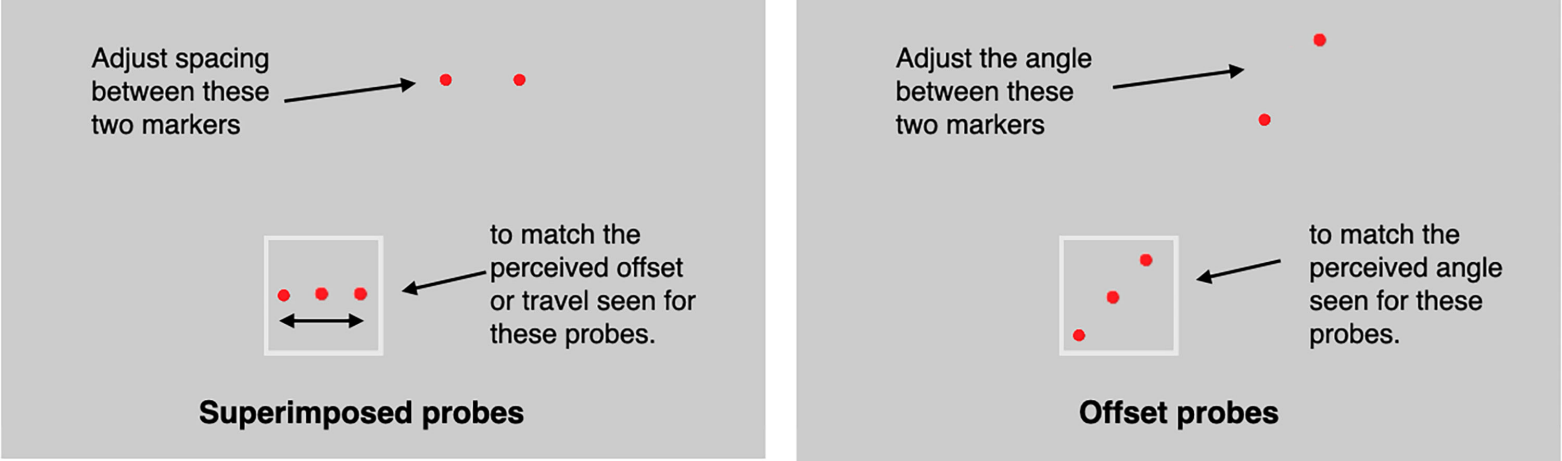
Matching procedure. The probes on the upper right were adjusted by the participants to match the apparent travel for the superimposed probes (left) or to match the apparent angle of motion for the vertically offset probes (right). Multiple flashes are shown in each frame here to indicate the perceived path but in the experimental display, only one probe was visible at a time as can be seen in Movie 4.

**Figure 5.**
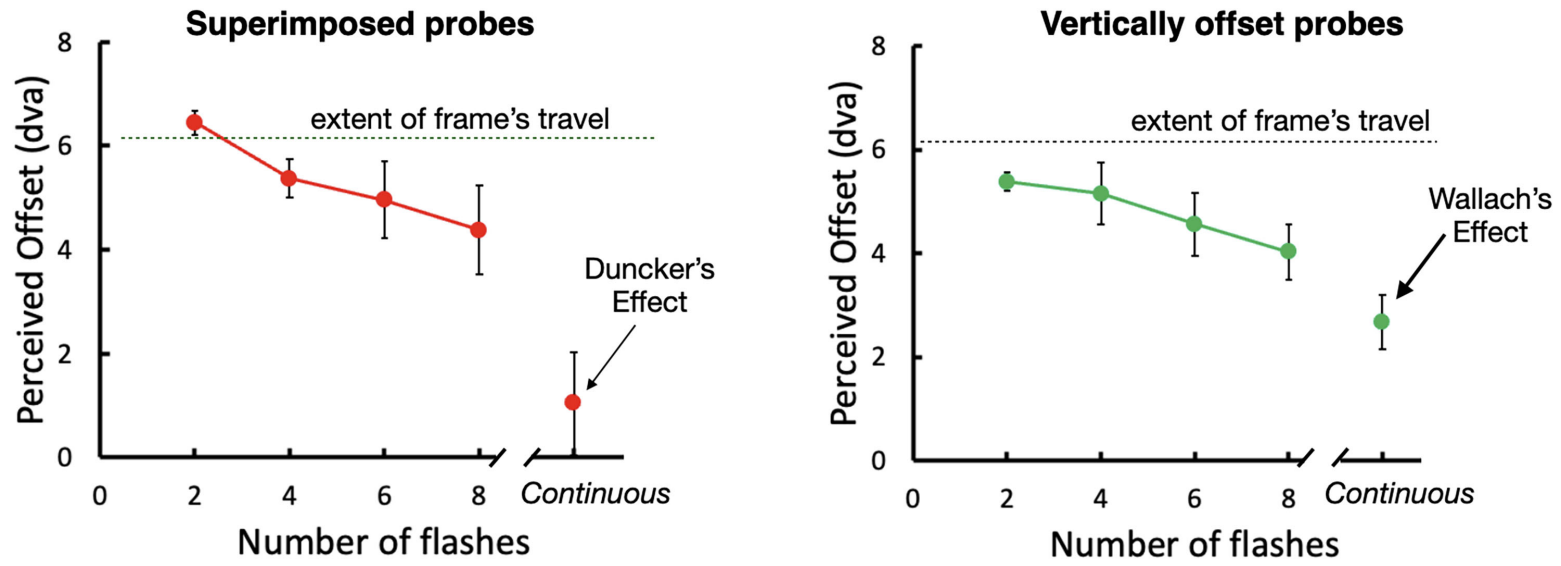
Perceived offset as a function of flicker rate of the probe. A perceived offset of 0 on the vertical axis indicates a veridical judgment with no illusion. (Left) Superimposed probes. The illusory offset starts near a 100% effect for low flicker rates and decreases as flicker rate increases. The continuous condition, equivalent to Duncker's (1929) stimulus, drops even more, showing a non-significant illusion. (Right) Vertically offset probes. The strength of the illusory shift again decreases as the flicker rate increases, but the continuous condition, equivalent to the Wallach et al. (1978) stimulus, still shows a significant illusion. Dva, degrees of visual angle.

In this paper, we explore the importance of the motion paths of the frame and the probes in producing the frame's effect.

## Experiment 1

We first compare the frame's effect on flashed tests (as in Özkan et al., 2021; Cavanagh et al., 2022) against its effect on a continuously moving test (as in Wallach et al., 1978). The frame is always in repetitive left to right motion (Figure 1, top; Movie 1). In addition, we vary the positions of the two flashed probes across a large range that includes the two locations that are aligned vertically on the screen and the two locations that are aligned within the frame. These different offsets between the two flashes will test whether the remarkable shifts (often 100% of the frame's travel) are a general property of the frame's effect on flashed probes, or are more limited. The 100% shift indicates that the probes’ positions are seen in frame coordinates and suggest a link to visual stability where objects do not appear to change position after an eye movement, despite their large change of position on the retina. If the scene that also shifts on the retina acts as a frame, then these conditions would also have effectively a 100% shift matched to the amplitude of the saccade. They would stay fixed in their position relative to the frame—the scene in this case. The critical test of the link to visual stability is the condition where the probes remain at the same location relative to the frame, but in a different location relative to the screen.

Similarly, with the continuously moving probe (Figure 1, bottom; Movie 2), its path angle will vary over a range that includes a vertical path on the screen (Wallach's original stimulus) and a vertical path relative to the frame. In each trial, observers could view the display as long as they wanted while adjusting a comparison stimulus on the top right of the screen to match the perceived offset or path that they saw for the flashed or continuous probes.

### Procedure

#### Participants

Eight individuals, including one of the authors, participated in the in-person experiments of this study (one female; age range, 22–75 years). All participants other than one author were naive to the purpose of this study and had normal or corrected-to-normal vision. Written, informed consent, approved by the Committee for the Protection of Human Subjects at Dartmouth College, was obtained from each participant prior to their experimental sessions.

#### Apparatus

All stimuli were generated an Apple Macintosh G4 computer with custom software written in C using the Vision Shell Graphics Libraries (Comtois, 2003). The display was presented on an LCD monitor with 60 Hz refresh rate and resolution of 800 × 600 pixels. The size of the display area was 40 × 30 degrees of visual angle (dva). Response adjustments were made with a track pad or mouse. Head movements were restrained with a chin rest and the viewing distance was 57 cm.

#### Stimuli

The screen was filled with a uniform mid-gray background and the lighter square frame had 50% contrast (Michelson) with the background. The frame size was 20 dva and the path length was 10 dva. The contour subtended 0.6 dva. The frame's motion path was centered horizontally on the display and the vertical center of the frame was 3.75 dva below the display's vertical midpoint to provide space for the measurement markers at the top right. The duration of the frame's motion was 166 ms and the pause at each end was 200 ms. The flashed probes were alternately red and blue, and the continuous probe was red. The probes were discs of 1.5 dva in diameter. In both conditions, two adjustment markers were present in the upper right of the display—10 dva horizontally from the screen center and 16.5 dva above the midpoint. They were discs with the same size and color as the probes.

### Procedure

Each trial began with a beep, after which the frame was present continuously and moved repeatedly back and forth horizontally.

#### Flashed probes

The red and blue probes flashed alternately each time the frame's motion reversed direction. They were presented for 33 ms, centered in the pause of the frame's motion at the end of each transit. The vertical offset between the red and blue probes was always 5 dva. The horizontal offset of the probes took 1 of 10 values on each trial: −5.0, −2.5, 0.0, 2.5, 5.0, 7.5, 10.0, 12.5, 15.0, and 17.5 dva. Here, a negative value indicates that the first, red probe was to the left of the second, blue probe and a positive value indicates that the red probe was to the right of the blue on the display. Relative to the frame, these same offsets were −15, 0.0, −12.5, −10.0, −7.5, −5.0, −2.5, 0.0, 2.5, 5.0, and 7.5 dva, respectively. Here, a negative value indicates that the red probe was to the left of the blue relative to the frame and vice versa for positive values.

#### Continuous motion

There was only one red probe, continuously present. It moved while the frame moved, always upward when the frame moved to the right and downward when it moved to the left and paused with the frame at the end of each transit. The vertical motion was always 5 dva. As the probe moved up and down it also moved horizontally by one of 3 values: 0, 5, or 10 dva on the display. Relative to the frame, these offsets were −10, −5, and 0 dva.

Participants were instructed to look around the display wherever they wanted as they made their setting, but to avoid fixating directly on a probe. Fixating on the probe allows it to be used as a reference point for the subsequent probe; for some observers, this weakens the effect. Moreover, in pilot experiments, we found that introducing any reference point on the display above or below the frame weakened the effect, because observers could then make the judgment based on the offset of the flash relative to that reference. That is why there is no fixation point in the display and the adjustment marker is off to the right and above the moving frame and probes. Using a mouse, they adjusted these two markers at the top right until their separation matched that of the flashed discs (or the angle of the continuous path). When they were satisfied with their match, they pressed the space bar, and the next trial began. There were four repetitions of each trial. The responses were self-paced, and participants could take a break at any time. The trials of the two conditions were randomly intermixed for a total of (13 × 4) 52 trials. Seven of the eight participants ran two sessions, one ran a single session. The experiment lasted about 15 minutes per session. The code, data, analyses, and individual plots are available at https://osf.io/wvpra/.

### Results

#### Flashed probes

Flashes that were in the same position on the screen (circled in green in Figure 2) were seen horizontally separated by approximately 10 dva, the distance the frame traveled, indicating almost full stabilization as if they were seen in the frame's coordinates and the frame were stationary. When the flashes were aligned in the same position relative to the frame but separated horizontally by 10 dva on the screen, they nevertheless appeared to be closer to vertically aligned than to veridical (70% stabilized, circled in magenta in Figure 2; see Movie 3).

#### Continuous probe

When the path of continuous motion was vertical on the screen (green circle in Figure 3), its path appeared rotated away from vertical, with the top end shifted 2.6 dva to the left from the bottom end (Movie 2). In comparison, for the flashed probes, the shift of the top flash relative to the bottom flash (the green circle in Figure 2) was 9.1 dva when the flashes were vertically aligned, an effect over three times as large.

### Conclusions

The effect of the frame for flashed tests was very large, almost 100% of the distance the frame traveled when the two flashes were aligned vertically on the screen. The flashes in this case are seen in the frame's coordinates, as if each is located relative to the frame at the moment it flashed: blue on the left and red on the right, despite being physically aligned one directly above the other on the screen. This large frame effect weakened when the probes were presented at the same location in the frame (magenta circle, Figure 2)—they did not look aligned as they would have if they were seen purely in frame coordinates but tilted away from that toward their veridical locations. The results for the continuous motion probe were very different. When the motion was purely vertical on the screen, it appeared tilted to the left (green circle in Figure 3). This is the classic induced motion result reported first with orthogonal frame and probe motion by Wallach et al. (1978). This classic effect is only approximately one-third the size of the displacement seen when the probes were flashed and aligned vertically on the screen.

The different offsets between the two probes in the flashed condition and between the motion end points in the continuous condition change the speed of the interflash and continuous motions of the probe by a factor of 2 or 3 between the vertical alignment (slowest) and the tilted alignments. However, at least in the case of the flashed probes, the speed (temporal offset between the flashes) was shown to have no effect on the apparent shift in location over a 64-fold range (Özkan et al., 2021), so we do not believe that the smaller speed changes here would have influenced these results.

Why does the continuous probe show such a reduced effect? There are two possible sources for the loss. First, the continuous probe provides more position information than the flashes and the uncertainty of the probe's location may, therefore, decrease over the presentation period. With a decrease in uncertainty, the probe may be less susceptible to the influence of the frame. Second, during the continued presence of the probe, the difference between its motion and the frame's motion may decrease the probe's grouping with the frame and allow it to be seen at least partly in the static reference frame of the background. The next experiment examines these two possibilities.

## Experiment 2

To understand the difference between continuous and flashed targets we varied the frequency from a single flash per transit of the frame up to continuously present (60 Hz). With more flashes, there will be more location samples to decrease positional uncertainty. However, the continuous, stationary case may be unique in supporting grouping with the background, whereas the flashed presentations do not favor such grouping. There were two conditions. In one, the multiple flashes were all superimposed so that, when the presentation was continuous, the probe was effectively stationary and corresponded to Duncker's (1929) original tests of induced motion (Movie 4, top right). In the second, the flashes were displaced vertically on the screen, moving upward from the bottom to the top (Movie 4, bottom row). When the probe was presented continuously, it moved orthogonally to the frame's motion, corresponding to the induced motion stimulus in Wallach et al. (1978). Probes were briefly flashed two, four, six, or eight times as the frame moved or continuously.

### Procedure

#### Participants

Four individuals, including one of the authors, participated in the in-person experiments of this study (one female; age range, 24–75 years). All participants had normal or corrected-to-normal vision. Written, informed consent, approved by the Glendon Psychology, Delegated Ethics Research Review Committee, York University, was obtained from each participant before their experimental sessions.

#### Apparatus

All stimuli were generated an Apple Macintosh G4 computer with custom software written in C using the Vision Shell Graphics Libraries (Comtois, 2003). The display was presented on an LCD monitor with a 60-Hz refresh rate and resolution of 1920 × 1080 pixels. The size of the display area was 60 × 33 dva. Response settings were made with a mouse. Head movements were restrained with a chin rest and the viewing distance was 57 cm.

#### Stimuli

The screen was filled with a uniform mid-gray background, and the lighter square frame had 75% contrast (Michelson) with the background. The frame size was 9.25 dva and the path length was 6.16 dva. The contour subtended 0.37 dva. The frame's motion path was centered 7.7 dva to the left of the display's horizontal midpoint and 6.16 dva below the display's vertical midpoint to provide space for the measurement markers at the top right. The duration of the frame's motion was 500 ms and the pause at each end was 66 ms. Probes were red discs of 0.925 dva in diameter, flashed for 33 ms centered in the 66-ms pause. In all conditions, two adjustment markers were present in the upper right of the display, 7.7 dva horizontally from the screen center and 10.3 dva above the midpoint. They were discs with the same size and color as the probes.

#### Procedure

Each trial began with a beep, after which the frame was present continuously and moved repeatedly back and forth horizontally. The flashed probes were presented between one and four times per transit of the frame so two, four, six, and eight times per left–right cycle (Movie 4). In the superimposed condition, all flashes were presented at the same location. In the offset condition, the probes moved vertically over a distance of 3.08 dva, moving up as the frame moved right and down as the frame moved left. Continuous probes were present through each trial, either stationary in one location for the superimposed condition or moving smoothly up and then back in the offset condition, covering 3.08 dva on each transit.

Participants were instructed to look around the display wherever they wanted as they made their setting, but to avoid fixating directly on a probe. Using a mouse, they adjusted the two markers at the top right until their separation matched the perceived separation between the outermost flashed probes or the angle of the continuous path (Figure 4). When they were satisfied with their match, they pressed the space bar, and the next trial began. There were four repetitions of each trial. The responses were self-paced, participants could take a break at any time. The trials of the two conditions (superimposed vs. offset) and five levels of flash (2, 4, 6, 8, and continuous) were randomly intermixed for a total of (10 × 4) 40 trials. The experiment lasted approximately 15 minutes.

### Analysis

The four settings (horizontal offsets) for each condition were averaged for each participant and the mean and standard error of the means across participants were calculated. The code, data, analyses, and individual plots are available at https://osf.io/wvpra/.

### Results

For the superimposed probes (Figure 5, left), the illusory shifts reached 100% of the frame's travel when only two flashes were presented, showing that the positions were seen in frame coordinates as if the frame were stationary, replicating the results of Experiment 1. The effect weakened with additional flashes and the simple position shift seen with two flashes was increasingly replaced by an induced motion. The illusory effect disappeared for continuous presentation of the probe, which for the superimposed condition appeared stationary, *t*(3) = 1.05, *p* = 0.19. The vertically offset probes showed a similar decrease in perceived offset with increasing flicker rates and a further drop when the presentation was continuous (Figure 5, right), but the illusion for continuous presentation remained significant, *t*(3) = 5.02, *p* = 0.008. This condition is equivalent to the stimulus in Wallach et al. (1978) and replicates the result for continuous vertical motion in Experiment 1 (Figure 3, green circle) with an illusory displacement of approximately one-third of the illusion seen for two flashes.

### Conclusions

The effect of the frame only disappeared for the stationary, continuously present probe. We suggest that the stationarity is a special case that allows the probe to group with the static background, cancelling any recovery of position relative to the frame. When the flashed tests were presented at the same location (Figure 5, left; Movie 4, top row), the frame's effect weakened with additional flashes, perhaps owing to the additional location information that was provided by each flash. However, the frame's effect with multiple flashes never dropped to the extent seen when the probe was continuous and stationary. When the probe was displaced vertically (Figure 5 right; Movie 4, bottom row), the frame's effect did weaken again with additional flashes but, importantly, it remained significant even when the probe was present continuously (Figure 5, rightmost datum point). This finding suggests that it is the stationarity that caused the loss of the illusion for the static probe (Figure 5, left), not the continuity. Nevertheless, when the continuous probe was moving, the frame's effect was weaker than for the flashed probes, suggesting that the continued presence of the probe during the frame's motion may allow it to group both with the frame and with the background.

## General conclusions

In the first experiment with flashed probes, we found first that the moving frame produced large spatial shifts of the perceived position that was, under some conditions, equal to the distance the frame traveled. For those conditions (Figure 2, left), the probes were seen in frame coordinates. However, this large effect size did not hold across the range of the physical offsets that were tested between the two probes. When the two probes were presented physically at the same location in each frame (and 10 dva apart on the screen), they should appear to be aligned vertically if their positions were determined relative to the frame. Instead, they had a significant tilt away from vertical toward their veridical positions. The frame was less effective in generating frame-based coordinates for the probes in this case. The shift that was seen was still approximately 70% of the maximum seen when the probes were physically aligned. This result suggests that the flashes are not always seen strictly in frame coordinates. Other factors may weaken the frame's effect for flashes more remote from the frame's borders like those presented at the center of the frame. This drop off with increasing distance from the contour to the flash is seen for other motion-induced position shifts like the flash grab (Cavanagh & Anstis, 2013), although, in that case, the drop off with distance is much more dramatic. The flash-lag effect may also play a role in counteracting the frame's influence when the flashes are near the middle of the frame. This lack of generality indicates that the link, if any, between the frame effect and visual stability may be complex.

For the continuous moving probes, the perceived shift was only approximately 30% of the shift that would be seen if the probe's location were perceived in frame coordinates rather than screen coordinates. Although the continuously moving probe must be grouping with the frame to some extent to produce this shift, the grouping was weaker than for the flashed probes. In the flash condition, the flashes occur simultaneously with the motion reversals so the synchronicity of the two transients should favor grouping the flash with the frame (common fate). With continuous presentation of the probe, there is less evidence for grouping with the frame as it becomes clear that the motions of the probe and frame are different. This may be seen as an ownership competition between the static background and the frame to see which would control the perceived location of the probe.

The second experiment explored the importance of continuity by comparing probes flashing at various rates (including continuous) that either remained at a fixed location or moved vertically. The results showed that the continuous, stationary probe was unique in having no significant position shift induced by the moving frame. In contrast, the continuously moving probe showed a significant perceived shift, although smaller than that seen for the flashed probes. This result indicated that continuity itself was not the source of the loss of effect for the stationary, continuous probe. It was the stationarity that eliminated the perceived shift most likely because the stationary probe grouped with the static background. As Duncker (1929) said for backgrounds that moved at higher rates, “If the [frame] is moved back and forth rapidly, the [probe] will often come to a standstill. This is because the [probe] loses, so to speak, its phenomenal relation to the [frame] as its background….When the immediate frame of reference fails, there occurs a spontaneous shift bringing the point into direct contact with the room environment where it becomes anchored” (p. 162).

These results leave us with two questions. First, how much motion is required in the continuous probe for it to decrease its grouping with the static background and begin grouping with the frame? Second, in our experiments, the continuously moving probe appears to group with both the frame and the background, but are there conditions under which a continuous probe will group only with the frame, ignoring the background? We present a rough outline of the answers here through informal observations (Cavanagh & Anstis, 2023) where we vary both frame and probe paths.

Movie 5 shows moving frames with continuous probes: on the left, the probe is stationary, whereas in the middle it has a small jittering motion. The stationary probe on the left shows little or no induced motion or position shifts. The frame appears to just slide back and forth over the stationary probe. In contrast, the jittering probe in the middle panel appears to move left and right within the frame, in the direction opposite to the frame, bouncing off its left and right borders. Even a slight motion of the probe seems to be enough to decrease the anchoring of the probe to the background and allow some induced motion relative to the frame. To further test the conjecture that the probe groups with the background when it has similar properties, a background is placed behind the frame and probe on the right in Movie 5 and the background and the probe jitter together. Here, although the probe is jittering as it was in the middle panel of Movie 5, it now loses its left-to-right induced motion. The frame may again appear to slide back and forth over the probe without affecting it, suggesting that the probe has grouped with the background rather than the frame. The source of the grouping between the probe and the background is likely their common motion: both are static together (Movie 5, left) or both are moving together (Movie 5, right). These demonstrations suggest that, once the probe is moving even slightly relative to the background, the link to the background is weakened, allowing the frame to influence the perceived position.

The results of Experiments 1 and 2 and these demonstrations suggest that a continuous probe must be moving to avoid grouping solely with a static background. However, the effect for continuously moving probes is only about 30% of that for flashed probes in Experiment 2, indicating that the continuous probe is still influenced by some partial grouping with the static background. Movie 6 demonstrates conditions under which the continuous moving probe can be seen to be more under the influence of the frame, independently of the background. The figure-8 in the moving frame looks much the same as the figure-8 in a stationary frame. In other words, we see strong frame-relative motion, independent of the background.

This emergence of the frame-relative motion is, of course, not new. Both Duncker (1929) and Johansson (1950) showed that path of a light at the edge of a rolling wheel, a cycloid, was seen quickly as circular once a light was attached to the center of the wheel. In this case as well, it is virtually impossible to see the cycloid once the path is seen to be circular relative to the wheel's center. The critical point may well be the complexity of the paths for the frame and the probe. When the paths are simple, as they are for the horizontal frame motion and vertical probe motion (Wallach et al. 1978; Movie 2), the probe's motion is not seen strictly in terms of its position in the frame, but it is a compromise between the motion relative to the frame and the motion relative to the static background. For more complex paths—the circular motion of the frame in Movie 6 and the figure-8 of its probe, or the circular path of the light at the wheel's edge for Duncker (1929) and Johansson (1950)—the probe's motion relative to the frame becomes dominant and the physical path becomes inaccessible.

In sum, we found again that flashes in a moving frame are displaced by the distance the frame travels. This replication of the frame stabilization effect (Duncker, 1929; Wong & Mack, 1981; Özkan et al., 2021; Cavanagh et al., 2022) did not hold up across all the possible offsets between the two flash locations that we tested. The induced displacement decreased to approximately 70% of the frame's displacement when the two flashes were in the center of the frame at both ends of its travel. This result raises questions concerning the link between this frame effect and visual stabilization. We also found the effect of the frame on a continuously moving probe to be approximately one-third the amplitude of the effect on two flashed probes, suggesting a competition for influence between the frame and the static background, much as Pressey (1967) had proposed in his assimilation theory for the Müller-Lyer illusion with gaps between the shafts and the arrowheads. The second experiment revealed that the absence of the frame's effect on the static continuous probe was because it grouped with its static background. Finally, informal observations (Movies 5 and 6) suggested that grouping the probe with either the frame or the background depended on factors of common motion and the complexity of the paths. Throughout the paper, we have assumed that grouping is the process that links either the frame or the background (or both) to the probe, affecting its perceived motion and position, but grouping is a vague term. There may be other processes involved that are not yet evident to us.

**Movie 5.** A continuous probe is presented in a frame moving left and right. Double click on the movies to open them in a separate window. Do not look directly at the probes in these movies, but somewhere outside each frame. (Left) A stationary probe shows little or no induced motion or position shift. (Middle) In contrast, with a slight jitter added to the probe, induced motion in the direction opposite to the frame's motion may be when not looking directly at the probe. (Right) When a textured background is added and the probe and the background jiggle together, the induced motion in the probe is reduced or eliminated so that it no longer appears to move left and right. These demonstrations are consistent with claim that the induced motion is lost when the probe groups with the background instead of the frame. Movie is available on the journal website.

**Movie 6.** Double click on the movies to open them in a separate window. (Left) A red disk follows a figure-8 path within a static textured rectangle. (Middle) The red disk moves along a figure-8 path with respect to the rectangle that itself moves in a circular path. The figure-8 path is still recognizable. (Right) The disk's path is a figure-8 plus a circle, following the same path relative to the screen as in the middle panel, but now without the rectangle. Here, the figure-8 curve is lost and the physical path, somewhat like an &, is seen. Movie is available on the journal website.
